# Recognizing an Incarcerated Gravid Uterus When the Cervical Canal Is Absent on Transvaginal Ultrasound

**DOI:** 10.7759/cureus.97996

**Published:** 2025-11-28

**Authors:** Asuka Okawa, Sho Tano, Mikako Inoue, Tatsuo Inamura, Satoru Katsuki, Kazuya Fuma, Seiko Matsuo, Takafumi Ushida, Kenji Imai, Tomomi Kotani, Hiroaki Kajiyama

**Affiliations:** 1 Department of Obstetrics, Nagoya University Hospital, Nagoya, JPN; 2 Department of Obstetrics and Gynecology, Kasugai Municipal Hospital, Kasugai, JPN; 3 Department of Obstetrics and Gynecology, Nagoya University Graduate School of Medicine, Nagoya, JPN; 4 Department of Obstetrics and Gynecology, Handa City Hospital, Handa, JPN; 5 Department of Obstetrics and Gynecology, Hamamatsu University School of Medicine, Hamamatsu, JPN

**Keywords:** incarcerated gravid uterus, obstetrics ultrasound, placenta previa, placenta previa mimic, retroverted uterus, uterine incarceration

## Abstract

Incarcerated gravid uterus (IGU) is an uncommon but consequential condition in which a retroverted gravid uterus remains trapped within the sacral hollow and may mimic placental location abnormalities, risking iatrogenic injury if unrecognized. We report a 39-year-old primigravida referred at 31 weeks of gestation with a diagnosis of complete placenta previa and uterine myomas. Transvaginal ultrasonography showed placental tissue interposed between the probe and fetus with nonvisualization of the endocervical canal; pelvic examination and transabdominal ultrasonography demonstrated marked rightward, cephalad cervical deviation and an apparent posterior low-lying placenta. Magnetic resonance imaging (MRI) supported uterine incarceration, likely exacerbated by a myoma. At 38+1 weeks, elective cesarean delivery was initiated via a 7-cm infraumbilical vertical skin incision to assess the feasibility of manual reduction; because reduction was difficult, the incision was extended transversely at the umbilical level to optimize exposure. Intraoperative ultrasonography identified the internal os and confirmed rightward, superior displacement of the lower uterine segment, guiding a transverse uterine incision. A healthy male infant weighing 2.892 g was delivered; the placenta was expelled easily, the uterus was repositioned without adhesiolysis, estimated blood loss was 1,113 mL, and no transfusion was required. This case underscores the value of coordinated transvaginal/transabdominal ultrasonography with MRI. It highlights nonvisualization of the endocervical canal between the transvaginal probe and presenting part as a practical red-flag sign of IGU that may aid differentiation from true placenta previa, prompt targeted anatomic assessment, and support safer operative planning.

## Introduction

Incarcerated gravid uterus (IGU) is an uncommon but clinically consequential condition in which a gravid, typically retroverted uterus becomes trapped within the sacral hollow instead of ascending into the abdomen. Reported frequency is approximately one in 3,000 pregnancies in contemporary reviews, with broader estimates up to one in 10,000 depending on case ascertainment and setting [1,2]. Physiologically, 6%-15% of uteri are retroverted in early pregnancy, and most spontaneously correct to an anteverted, abdominal position by around 14-16 gestational weeks as the fundus rises out of the pelvis [3]. In some cases, however, the retroverted gravid uterus fails to ascend, becoming trapped within the sacral hollow and resulting in incarceration. This altered configuration can compress the bladder and rectum, leading to urinary symptoms, pelvic discomfort, or none.

Risk factors include uterine myomas, pelvic adhesions, and congenital uterine anomalies; however, IGU can occur without symptoms, contributing to underrecognition. Misdiagnosis is frequent because clinical signs are nonspecific (e.g., urinary complaints, pelvic pain, or none), and the distorted pelvic anatomy can mimic other entities on imaging. Notably, IGU may simulate placental location abnormalities (e.g., complete placenta previa or low-lying placenta), potentially driving inappropriate delivery planning if the diagnosis is missed [4,5].

Targeted imaging is pivotal. Sonographic clues include marked deviation/elongation of the cervix and an apparent posterior “low-lying” placenta when the lower uterine segment is actually displaced cephalad. Magnetic resonance imaging (MRI) provides complementary anatomic delineation and can secure the diagnosis when ultrasound is equivocal, thereby mitigating maternal-fetal risk through informed operative planning [4,6].

Management strategies depend on gestational age, symptoms, and the feasibility of reduction. Early recognition may allow conservative measures (knee-chest positioning, bladder emptying, and gentle manual reduction), while persistent or late-diagnosed cases warrant carefully planned cesarean delivery with attention to altered relationships of the bladder, cervix, and lower uterine segment [4,7,8].

Here, we report a third-trimester case of IGU that closely mimicked complete placenta previa on initial assessment. Importantly, our case highlights a practical sonographic clue: absence of a visible endocervical canal interposed between the transvaginal probe and the presenting fetal part in the setting of apparent posterior placentation. This case underscores key diagnostic pitfalls and practical steps to avoid iatrogenic complications when IGU is suspected.

## Case presentation

**Figure 1:**
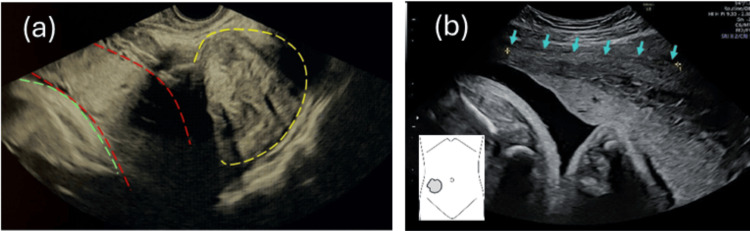
Ultrasonographic findings (a) Transvaginal ultrasonography showing the placenta (outlined in red dashed lines), the fetal buttocks (green dashed lines), and a uterine myoma in the pouch of Douglas (yellow dashed lines). (b) Transabdominal ultrasonography with the probe positioned on the patient's right lower abdomen, as illustrated in the schematic, showing marked ventral elongation of the cervix (blue arrows) with the placenta attached to the posterior uterine wall and its edge 1.8 cm away from the cervical os

**Figure 2:**
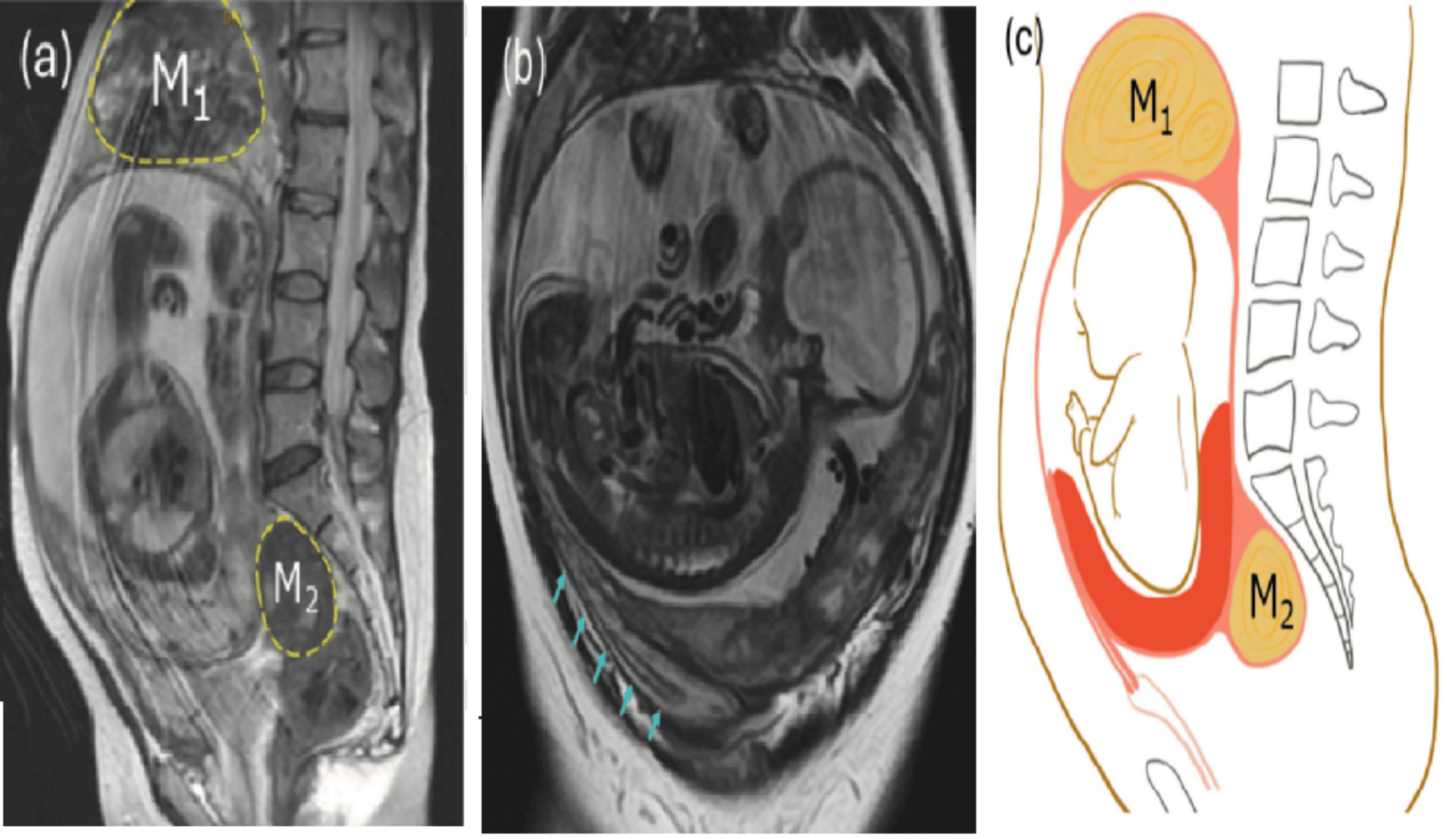
Imaging and schematic of the anatomical findings (a) MRI showing uterine myomas measuring 20 and 10 cm in diameter located at the cranial side of the uterus and the caudal side of the sacral promontory, respectively (outlined in yellow dashed lines). (b) MRI showing marked ventral elongation of the cervix in the right cephalad direction. The cervical line is indicated by blue arrows. (c) Schematic of the anatomical findings M_1_: myoma 1; M_2_: myoma 2; MRI: magnetic resonance imaging Image credit: Figure 2c is an original image created by the authors Sho Tano and Mikako Inoue

A 39-year-old primigravida with no significant medical history was referred to our hospital at 31 weeks of gestation with diagnoses of complete placenta previa and uterine myomas. Transvaginal ultrasonography showed a 10-cm mass in the pouch of Douglas and placental tissue interposed between the ultrasound probe and the fetus; the cervical canal was not visualized (Figure 1a). Pelvic examination demonstrated marked rightward deviation of the cervix. Transabdominal ultrasonography demonstrated pronounced rightward, cephalad elongation of the cervix, with the placental edge 1.8 cm from the cervical canal (Figure 1b). On this basis, IGU with a posterior low-lying placenta was suspected. MRI suggested that the incarceration was attributable to a myoma (Figure 2).

**Figure 3:**
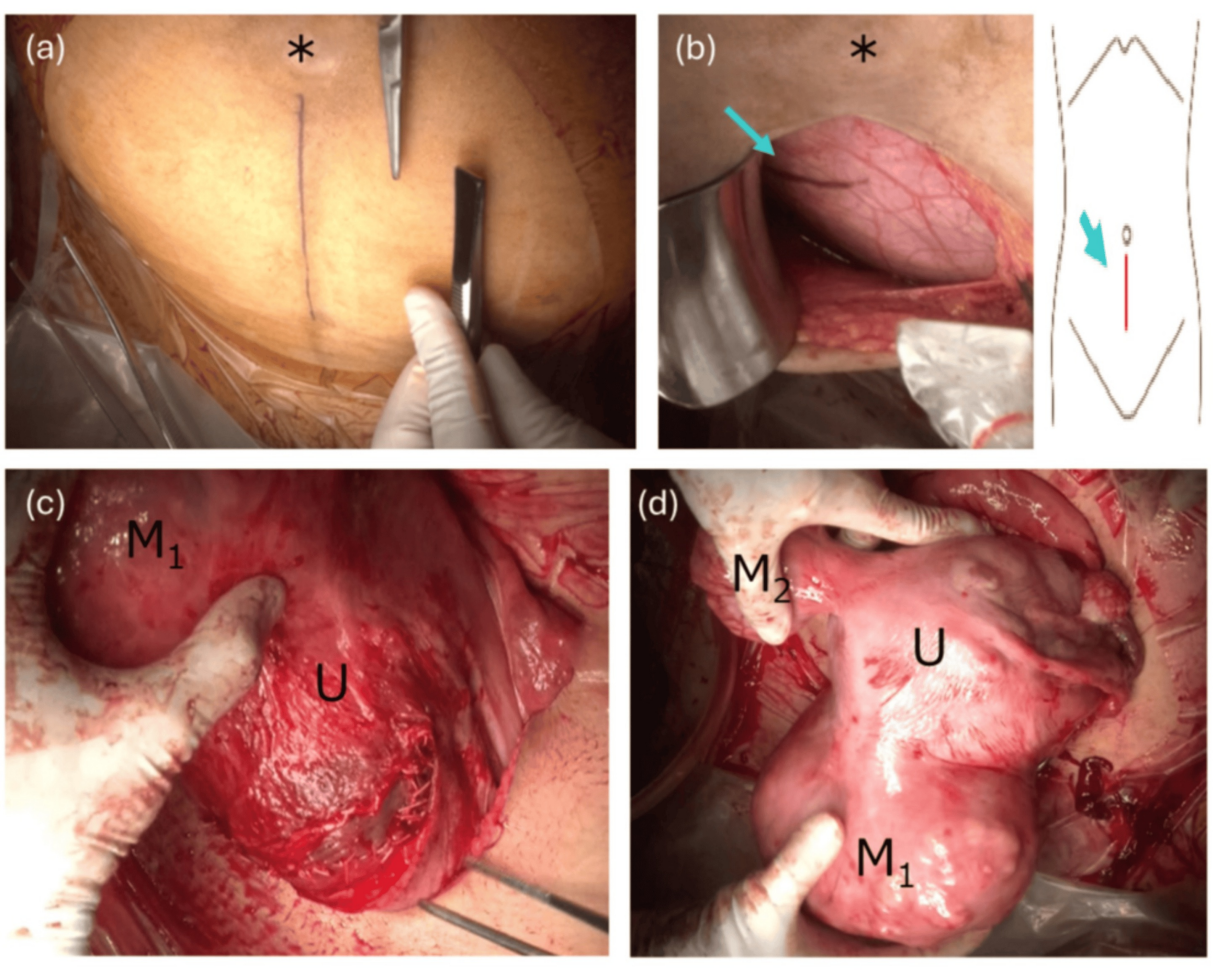
Intraoperative findings, incision strategy, and uterine anatomy (a) Before incision, the planned 7-cm infraumbilical vertical skin incision is marked. ＊indicates the umbilicus. (b) After initial entry and assessment, the internal os (blue arrow) is identified with ultrasonography (schematic at right). ＊indicates the umbilicus. (c) A transverse uterine incision at the lower uterine segment was achieved. (d) Exteriorized uterus (U) showing displacement/distortion with two myomas (M₁, M₂): no adhesions to surrounding structures U: exteriorized uterus; M_1_: myoma 1; M_2_: myoma 2

An elective cesarean delivery via a vertical abdominal incision was performed at 38＋1 weeks of gestation. We began with a 7-cm infraumbilical vertical skin incision and first assessed whether the incarcerated uterus could be manually reduced. Because manual reduction proved difficult, the skin incision was extended transversely at the level of the umbilicus to optimize exposure and proceed with the operation. Intraoperative ultrasonography was used to identify the internal cervical os, confirming that the lower uterine segment was displaced to the right and superiorly, concordant with preoperative imaging (Figure 3 and Video 1). A transverse uterine incision was made, and a healthy male infant weighing 2.892 g was delivered. The placenta was expelled without difficulty, and the uterus was repositioned; no adhesions to surrounding structures were noted. The total estimated blood loss was 1,113 mL, and no blood transfusion was required.

**Video 1:** Intraoperative findings, incision strategy, and uterine anatomy We began with a 7-cm infraumbilical vertical skin incision and first assessed whether the incarcerated uterus could be manually reduced. Because manual reduction proved difficult, the skin incision was extended transversely at the level of the umbilicus to optimize exposure and proceed with the operation. Intraoperative ultrasonography was used to identify the internal cervical os, confirming that the lower uterine segment was displaced to the right and superiorly. A transverse uterine incision was made, and a healthy male infant weighing 2.892 g was delivered. The placenta was expelled without difficulty, and the uterus was repositioned; no adhesions to surrounding structures were noted

## Discussion

Although urinary dysfunction and lower abdominal pain are frequently reported in IGU, 8%-21% of patients, our case included, are asymptomatic, which contributes to delayed or missed diagnoses [1]. The anatomic distortion in IGU readily confounds imaging interpretation and can be mistaken for placental location abnormalities; indeed, approximately 39% of misclassifications are attributed to placental disorders [1]. In this context, our case underscores a practical sonographic clue: absence of a visible endocervical canal interposed between the transvaginal probe and the presenting fetal part in the setting of apparent posterior placentation. Prior reports have emphasized cervix deviation/elongation and seemingly posterior low-lying placenta [4,5]; the present observation complements these features as a simple, actionable “red-flag” for suspecting IGU. Table 1 compares IGU and placenta previa across clinical presentation, imaging hallmarks, and operative planning, highlighting our proposed red-flag sign of endocervical canal nonvisualization.

**Table 1:** Comparative summary of IGU vs. true placenta previa ^†^Proposed red-flag sign from the present case IGU: incarcerated gravid uterus; PAS: placenta accreta spectrum; CS: cesarean section

Feature	IGU	Placenta previa
Symptoms	Often urinary complaints (retention, frequency) or pelvic discomfort; may be asymptomatic	Painless third-trimester bleeding; may be recurrent; no urinary retention pattern
Cervix	Deviated and elongated; may be difficult to reach	Usually midline/accessible unless bleeding limits exam
Cervical canal	Nonvisualization of the endocervical canal between the probe and the placenta/fetal head^†^	Canal typically visible; placenta overlies/approaches internal os
Lower uterine segment	Lower uterine segment displaced cephalad and to one side; cervix “ascends” away	Lower uterine segment position normal; placenta encroaches on/over internal os
Bladder	Often displaced anterior-superior	Normal location
Delivery plan	If unreduced, planned CS with imaging-guided mapping (consider vertical skin incision for exposure; intraoperative ultrasound to locate internal os/lower uterine segment)	Planned CS if persistent placenta over/near os; multidisciplinary planning if PAS suspected

Coordinated interpretation of transvaginal and transabdominal ultrasonography, supplemented by MRI, enabled preoperative delineation of the rightward, cephalad displacement of the lower uterine segment and the anterior-superior shift of the bladder in our patient. Ultrasonography is well-suited to dynamically trace the cervical trajectory and locate the true lower uterine segment, while MRI increases diagnostic confidence when sonography is equivocal and informs surgical planning. Notably, intraoperative ultrasound guidance facilitated real-time identification of the internal os and safe placement of a transverse uterine incision despite distorted anatomy, mirroring recommendations from prior reports [4,5,9].

Management should be tailored to gestational age, symptom burden, and the feasibility of uterine reduction. When recognized by mid-gestation and symptoms are manageable, conservative measures, knee-chest positioning, bladder emptying, and gentle manual reduction, may be attempted first [4,7]. In contrast, persistent cases into the third trimester or failed reductions warrant planned cesarean delivery that anticipates altered pelvic relationships. In our case, preoperative imaging prompted a vertical midline abdominal approach, followed by intraoperative ultrasound confirmation of the deviated lower uterine segment and a transverse uterine incision, which together supported hemostasis and minimized risk to adjacent organs.

Because the bladder tends to be displaced anteriorly and superiorly, the cervix is deviated and elongated, and the lower uterine segment is shifted cephalad, routine reliance on expected landmarks risks bladder or cervical injury in IGU. Preoperative mapping, ultrasonography with MRI as needed, and intraoperative ultrasound verification of the incision line are prudent steps to enhance safety [4]. Unrecognized IGU has also been associated with uterine rupture during attempted vaginal delivery; therefore, delivery plans should be reevaluated promptly when IGU is suspected [4].

This report highlights three pragmatic lessons: 1) even when imaging suggests placenta previa, attention to nonvisualization of the endocervical canal between the transvaginal probe and the presenting part can trigger consideration of IGU; 2) combining transvaginal/transabdominal ultrasound with MRI yields a clear anatomic map for operative planning; and 3) intraoperative ultrasound can guide a safe uterine incision in a distorted pelvis. The proposed “canal nonvisualization” sign complements established clues (cervical deviation/elongation and apparent posterior low-lying placenta) [4,5] and may aid differentiation from true placenta previa while avoiding unnecessary or hazardous maneuvers.

As a single-case report, our observation regarding canal nonvisualization requires validation. Its sensitivity, specificity, and interreader reproducibility remain to be determined in larger series. Nonetheless, given the nonspecific symptoms and frequent imaging pitfalls of IGU, introducing a simple, easily checkable sonographic checkpoint has practical clinical value and may reduce iatrogenic risk.

## Conclusions

IGU is easily missed because symptoms are nonspecific and anatomy is distorted on routine views. Our case proposes a practical checkpoint, the nonvisualization of the endocervical canal between the transvaginal probe and the presenting part in apparent posterior placentation-as a red-flag sign to prompt timely consideration of incarceration and reduce iatrogenic risk.
